# Assessment of community awareness and attitude about the use of GLP 1 agonists as a treatment for obesity in Saudi Arabia: Cross-sectional study

**DOI:** 10.1097/MD.0000000000046957

**Published:** 2026-01-09

**Authors:** Amal Khaleel Abualhommos, Maitham Abdullah Al Hawaj, Sharifa Yousef Alrasheed, Reem Abdulrahman Alessa, Alzahra Yousef Alhussain, Rouaa Adel Alabdulkareem, Raghad Ibrahim Abdulaziz Alzamil

**Affiliations:** a Pharmacy Practice Department, Clinical Pharmacy College, King Faisal University, Alhasa, Saudi Arabia.

**Keywords:** attitude, awareness, GLP 1 agonists, obesity, Saudi Arabia

## Abstract

Glucagon-like peptide-1 receptor agonists (GLP-1 RAs) are a commonly used class of antidiabetic medications in the treatment of type 2 diabetes mellitus and obesity management, and it is important to understand public awareness and attitudes toward GLP-1 RAs. This study aims to identify the level of awareness and knowledge about GLP-1 RAs in a heterogeneous population. This is an online survey study that was conducted in Saudi Arabia between December 2024 and January 2025 to examine community awareness and attitudes regarding the use of GLP-1 agonists as a treatment for obesity in Saudi Arabia. The questionnaire tool for this study was developed based on an extensive literature review. Multiple logistic regressions were performed to identify the predictors of knowledge about GLP-1 agonists. A total of 398 participants were included in the analysis. Regarding the GLP-1 enhancers, the majority (289, 72.6%) had heard of them before participating in the online survey. According to our participants, the most familiar one was mounjaro (262, 65.8%). Social media was the most common source (227, 57.0%). The majority reported using GLP-1 enhancers for weight reduction (319, 80.4%). Common side effects included loss of appetite (276, 69.3%), vomiting (196, 49.2%), and abdominal pain (188, 47.2%). The most frequently mentioned contraindications were pregnancy and lactation (295, 74.1%), pediatric age group (204, 51.3%), and kidney conditions (171, 43.0%). Individuals with an income between 5000 to 10,000 Saudi Arabian riyal (SAR) had significantly less knowledge of GLP-1 enhancers compared to those earning <5000 SAR (OR = 018, 95% CI: 0.03–0.99, *P* = .04). Additionally, individuals who had previously used or were currently using GLP-1 enhancers were significantly more likely to have better knowledge (OR = 6.56, 95% CI: 1.82–23.66, *P* = .004). Participants were moderately to highly aware of GLP-1 enhancers. Most people took these drugs to lose weight, with side effects including appetite loss, vomiting, and abdominal pain. Lactation, pregnancy, pediatric usage, and kidney problems were common contraindications. Lower-income groups knew more about GLP-1 enhancers than higher-income ones, and former or present users knew the most. Future studies should examine income-related knowledge gaps and social media’s educational potential. GLP-1 enhancers’ long-term safety, efficacy, and awareness across varied populations must be assessed in longitudinal research.

## 1. Introduction

Diabetes mellitus (DM) is considered a potentially life-threatening medical condition, associated with a range of severe chronic and acute outcomes.^[1–3]^ Glucagon-like peptide-1 receptor agonists (GLP-1 RAs) are now an anchor drug class in the treatment of type 2 DM and obesity management.^[4]^ With their mechanism of action, they cause effects through the secretion of insulin, suppression of glucagon release, and increased satiety, leading to improved glycemic control along with significant weight loss.^[5]^ Given their growing role in metabolic disease management, it is important to understand public perception, usage patterns, and attitudes toward GLP-1 RAs in a bid to ensure maximum patient compliance and education.

Although clinically well understood, knowledge gaps also exist for GLP-1 RAs regarding their indications, side effects, and contraindications.^[6]^ Previous studies have delineated variations in levels of awareness as a function of socio-demographic factors, access to health services, and media exposure.^[7]^ Patient adherence to GLP-1 RAs has also been previously influenced by self-perceived efficacy, probability of side effects, and patient trust in healthcare providers.^[8]^ Therefore, consideration of determinants of attitude and knowledge towards such drugs is essential in creating educationally focused interventions as well as enhancements in treatment outcomes.

According to the General Authority for Statistics in Saudi Arabia, the obesity rate in 2024 reached 23.1% among individuals aged 15 years and older. Additionally, 45.1% of those individuals are classified as overweight.^[9]^ GLP-1 RAs are now considered a cornerstone in effective obesity management protocols.^[10]^ This is supported by wide-ranging evidence from randomized controlled trials and meta-analyses.^[11]^ The use of GLP-1 RAs is associated with weight loss of 4 to 16 kg.^[11,12]^ Moreover, treatment guidelines recommend these medications as first-line therapy among various patient groups, specifically those with comorbid conditions.^[13]^

This study aims to identify the level of awareness and knowledge about GLP-1 RAs among the general public in Saudi Arabia. The specific objectives were to identify sources of information considered most important and to investigate the determinants of differences in knowledge. By conducting demographic characteristics analysis, history of exposure to drugs, and information channels of dissemination, this study presents various insights regarding patient attitudes to GLP-1 RAs. The findings will inform public health interventions aimed at increasing awareness and promoting informed decision-making regarding the use of these drugs.

## 2. Methods

### 2.1. Study design

This is an online survey study that was conducted in Saudi Arabia between December 2024 and January 2025.

### 2.2. Study population and sampling technique

Saudi Arabian males and females from the general public aged between 18 and 65 made up the study’s population. Males and females between the ages of 18 and 65 who reside in Saudi Arabia were eligible to participate. Saudi and non-Saudi individuals who reside outside Saudi Arabia and are younger than 18 or older than 65 were excluded.

A convenience sampling technique was used to recruit the participants for this study. Social media platforms, including WhatsApp and Facebook, were used to circulate the questionnaire link and recruit participants. The questionnaire link included an invitation letter highlighting the study’s aims and inclusion criteria. Participants who met the inclusion criteria were asked to participate in the study.

### 2.3. Questionnaire tool

The questionnaire tool for this study was developed based on an extensive literature review.^[14,15]^ The questionnaire was created in the Arabic language. The questionnaire examined participants’ characteristics (age, gender, education level, income, body mass index, family member working in healthcare, and having a history of DM). Additionally, the questionnaire examined familiarity with GLP-1 types, sources of information, utilization patterns, side effects, and contraindications, and awareness, perceptions, and concerns regarding GLP-1 enzyme enhancers. The questionnaire instrument was formatted in a multiple-choice format. The knowledge score had 4 items; the median summation of the items was used as a cutoff point for categorizing the score into poor and good knowledge.

The external validity of the questionnaire tool was checked by 2 clinical pharmacists. They confirmed that the questionnaire tool covered the study objectives with no editing needed. This was followed by a pilot study on 25 participants who met the inclusion criteria for this study. The participants were asked about the questionnaire items, and they confirmed that they were clear and understandable.

### 2.4. Ethical approval

Ethical approval for this study was obtained from the Research Ethics Committee at King Faisal University (KFU-REC-2024-NOV-ETHICS2880). All participants provided their consent before participating in the study.

### 2.5. Data analysis

Categorical variables were presented in terms of frequency and percentage. A multiple logistic regression was performed with knowledge as the dependent variable; demographic and other variables were utilized as independent variables. The findings of the regression analysis were presented as odds ratios along with their corresponding 95% confidence interval. The level of significance was defined as a *P*-value <.05. The data were analyzed using Statistical Package for the Social Sciences (SPSS), version 29.

## 3. Results

A total of 398 participants were included in the analysis. The majority of participants (221, 55.5%) were aged between 19 and 39 years, followed by those between 40 and 59 years (124, 31.2%). The majority of participants (307, 77.1%) were females, with 91 males (22.9%). Regarding monthly income, 155 (38.9%) had a monthly income between 5000 and 10,000 Saudi Arabian riyals (SAR), and 125 participants (31.4%) earned <5000 SAR monthly. Most participants (261, 65.6%) had a bachelor’s degree, followed by 99 participants who had only a high school degree (24.9%). Additional demographic characteristics are provided in Table 1. Regarding the GLP-1 enhancers, the majority (289, 72.6%) had heard about the term before participating in the online survey. According to our participants, the most familiar one was Mounjaro (262, 65.8%), followed by Ozempic (237, 59.5%), while only 68 participants (17.1%) had heard about Saxenda. More details in Table 2.

**Table 1:** Demographic characteristics of the participants.

Demographic characteristics	N	%
Age (yr)		
<18	21	5.3
19–39	221	55.5
40–59	124	31.2
60–64	32	8.0
Gender		
Female	307	77.1
Male	91	22.9
Education level		
Less than high school	17	4.3
High school	99	24.9
Bachelor	261	65.6
Higher than bachelor	21	5.3
Income (SAR)		
<5000	125	31.4
5000–10,000	155	38.9
>10,000	118	29.6
BMI		
<18	39	9.8
18–24.9	135	34.1
25–30	101	25.5
30–35	67	16.9
>35	54	13.6
Family member working in healthcare		
No	229	57.5
Yes	169	42.5
Diabetes mellitus history		
No	355	89.2
Yes	43	10.8

BMI = body mass index, SAR = Saudi Arabia riyal.

**Table 2:** Familiarity with GLP-1 types.

	N	%
Did you hear before about GLP-1 enhancer		
No	109	27.4
Yes	289	72.6
Saxenda		
No	330	82.9
Yes	68	17.1
Mounjaro		
No	136	34.2
Yes	262	65.8
Ozempic		
No	161	40.5
Yes	237	59.5

GLP-1 = glucagon-like peptide-1.

Among the sources of information, social media was the most common (227, 57.0%), followed by friends and family (153, 38.4%) and healthcare professionals (105, 26.4%). The majority reported using GLP-1 enhancers for weight reduction (319, 80.4%) and glucose control (212, 53.3%), while fewer used them for reducing the risks of obesity related disease (117, 29.4%). Common side effects included loss of appetite (276, 69.3%), vomiting (196, 49.2%), and abdominal pain (188, 47.2%). The most frequently mentioned contraindications were pregnancy and lactation (295, 74.1%), pediatric age group (204, 51.3%), and kidney conditions (171, 43.0%). More details in Table 3.

**Table 3:** Sources of information, uses, side effects, and contraindications.

	N	%
Source of information		
Social media		
No	171	43.0
Yes	227	57.0
Internet		
No	324	81.4
Yes	74	18.6
Healthcare professional		
No	293	73.6
Yes	105	26.4
Television		
No	368	92.5
Yes	30	7.5
Friends and family		
No	245	61.6
Yes	153	38.4
I didn’t hear it before		
No	344	86.4
Yes	54	13.6
Uses of GLP-1 enhancer		
Reducing weight		
No	78	19.6
Yes	319	80.4
Glucose control		
No	186	46.7
Yes	212	53.3
Reducing risk of obesity disease		
No	281	70.6
Yes	117	29.4
Kidney		
No	22	100.0
Yes	0	0.0
Cardiovasular System		
No	397	99.7
Yes	1	0.3
Side-effect		
Loss of appetite		
No	122	30.7
Yes	276	69.3
Constipation		
No	275	69.1
Yes	123	30.9
Diarrhea		
No	277	69.6
Yes	121	30.4
Abdominal pain		
No	210	52.8
Yes	188	47.2
Vomiting		
No	202	50.8
Yes	196	49.2
Headache		
No	232	58.3
Yes	166	41.7
Contraindications		
Diabetes mellitus		
No	341	85.7
Yes	57	14.3
Pregnancy and lactation		
No	103	25.9
Yes	295	74.1
Children		
No	194	48.7
Yes	204	51.3
Stomach		
No	216	54.3
Yes	182	45.7
Pancreatitis		
No	221	55.5
Yes	177	44.5
Kidney		
No	227	57.0
Yes	171	43.0
Heart		
No	265	66.6
Yes	133	33.4

GLP-1 = glucagon-like peptide-1.

A total of 175 participants (44.0%) reported being fully aware of this type of medication and its uses, and a higher percentage (272, 68.3%) claimed to know about their potential side effects. Trust in healthcare providers as a source of information was high, with 312 participants (78.4%) agreeing they are trustworthy. Additionally, 239 participants (60.1%) felt confident in adhering to the treatment plan, and 297 individuals (74.6%) recognized the importance of regular follow-up appointments (Table 4).

**Table 4:** Awareness, perceptions, and concerns regarding GLP-1 enzyme enhancers.

	N	%
I am fully aware of GLP-1 enzyme enhancers from the stomach and their uses.		
Not agreed	223	56.0
Agreed	175	44.0
I believe that GLP-1 enzyme enhancers from the stomach are effective only for treating obesity.		
Not agreed	258	64.8
Agreed	140	35.2
I am aware of the potential side effects of these medications.		
Not agreed	126	31.7
Agreed	272	68.3
I trust the information I receive about GLP-1 enzyme enhancers from my healthcare provider (pharmacist, doctor, etc).		
Not agreed	86	21.6
Agreed	312	78.4
I feel confident in my ability to adhere to the treatment plan for GLP-1 enzyme enhancers.		
Not agreed	159	39.9
Agreed	239	60.1
Regular follow-up appointments are important for managing my treatment with GLP-1 enzyme enhancers.		
Not agreed	101	25.4
Agreed	297	74.6
I have concerns about the safety of GLP-1 enzyme enhancers.		
Not agreed	98	24.6
Agreed	300	75.4
I have thoroughly researched GLP-1 enzyme enhancers before starting the treatment plan or using them for obesity management.		
Not agreed	182	45.7
Agreed	216	54.3

GLP-1 = glucagon-like peptide-1.

Significant associations were observed between income and prior use of these medications. Individuals with an income between 5000 to 10,000 had significantly less knowledge of GLP-1 enhancers compared to those earning <5000 SAR (OR = 018, 95% CI: 0.03–0.99, *P* = .04). Additionally, individuals who had previously used or were currently using GLP-1 enhancers were significantly more likely to have better knowledge (OR = 6.56, 95% CI: 1.82–23.66, *P* = .004). Additional details about factors that influenced participants’ knowledge are provided in Table 5.

**Table 5:** Factors associated with knowledge: a multiple logistic regression.

	OR (95% CI)	*P*-value
Age		
<18	Reference	
19–39	0.54 (0.07–4.22)	.559
40–59	0.12 (0.01–2.01)	.139
60–64	0.31 (0.02–5.23)	.417
Gender		
Female	Reference	
Male	0.53 (0.12–2.39)	.408
Education level		
Less than high school	Reference	
High school	1.50 (0.10–22.04)	.768
Bachelor	1.05 (0.06–17.21)	.971
Higher than Bachelor	0.80 (0.02–27.33)	.904
Income		
<5000	Reference	
5000–10,000	0.18 (0.03–0.99)	.049
>10,000	0.71 (0.14–3.75)	.690
Family member in health system		
No	Reference	
Yes	3.04 (0.85–10.87)	.088
Are you using or did you use		
No	Reference	
Yes	6.56 (1.82–23.66)	.004
Constant	0.04 (0.00–0.00)	.016

CI = confidence interval, OR = odds ratio.

## 4. Discussion

The findings of this study provide significant insights into the awareness, usage patterns, side effects, and contraindications associated with GLP-1 RAs. A substantial proportion of participants (72.6%) reported having prior knowledge of GLP-1 enhancers, with Mounjaro being the most recognized drug (65.8%), followed by Ozempic (59.5%). On the other hand, Saxenda was the least familiar and relatively unknown (17.1%). This discrepancy reflects a variation in awareness among the overall population around particular GLP-1 RAs, perhaps due to marketing, physician advice, and online discussion. Findings are aligned with investigations proving variations in awareness based on media consumption and consultations with doctors.^[7,16]^ Various other research studies have established that newer agents, e.g., semaglutide, have been more favored in light of their demonstrated advantages in clinical trials and better weight loss performance.^[17,18]^

The most frequent reasons for using GLP-1 RAs among the participants were weight loss (80.4%) and control of blood sugar (53.3%), followed by a smaller group (29.4%) who said they used them to avoid obesity-related diseases. The findings align with previous studies that indicated GLP-1 RAs are prescribed primarily because of their action to reduce blood sugar and help with weight loss.^[5]^ Nonetheless, the proportionally lower percentage of patients using these drugs for obesity-related disease prevention indicates a shortfall in patient education on their general benefits beyond diabetes control.^[19]^

Side effects listed aligned with those reported during clinical trials. Most frequent adverse effects noted were loss of appetite (69.3%), vomiting (49.2%), and abdominal pain (47.2%). All such side effects that are gastrointestinal in origin are common and have been regarded by many as the chief determinant affecting patient compliance with the treatment.^[20]^ Psychiatric side effects such as mood change and depression were also reported and have raised concerns in recent research. These are especially prevalent with Ozempic (semaglutide), and point to the need for further study.^[21]^

Contraindications were also documented in bold, the highest frequency being pregnancy and lactation (74.1%), pediatric age group (51.3%), and kidney disease (43.0%). The findings concur with clinical practice recommendations that contraindicate the use of GLP-1 RAs in such groups due to safety concerns.^[22]^ More recent meta-analyses demonstrate that GLP-1 RAs have protective renal effects in diabetes patients, and contraindications should therefore be assessed individually.^[23]^

The study also identified a strong association between levels of knowledge and demographic factors. Of particular concern was that participants within the income group of 5000-10,000 SAR were identified as having significantly lower knowledge levels about GLP-1 enhancers compared to participants whose income level was <5000 SAR (OR = 0.18, 95% CI: 0.03–0.99, *P* = .04). The negative association could be a sign of disparities in access to health information or differences in the patterns of health information seeking across income levels. This could be due to the heavy exposure to diabetes education in the public healthcare centers compared to the private sector.^[24,25]^ Further, multiple factors might affect GLP-1 RAs-related knowledge, such as education level, disease duration, and participants’ self-management behavior.^[26]^ This was confirmed by multiple studies from Saudi Arabia, which showed that information sources and education level were important predictors of diabetes knowledge. Previous studies showed that social media platforms have improved public awareness of GLP-1 RAs as a first-line treatment for obesity, with demonstrated effectiveness in weight loss.^[27]^ This could have led to the better awareness and comprehension related to these medications.

Previous research has also indicated socioeconomic factors as being determinative in medication awareness and availability.^[28]^ Patients who were previous users or were currently using GLP-1 RAs were also much more likely to have enhanced knowledge about these medications (OR = 6.56, 95% CI: 1.82–23.66, *P* = .004). This discovery points toward the increased experience with the drugs being associated with higher experience of their impact, use, and risk factor awareness, signifying the presence of experiential learning as part of medical literacy. These observations emphasize the need for educational interventions through proper educational strategies, ensuring that understanding and awareness with respect to GLP-1 RAs increase, especially within lower socioeconomic groups, as well as patients without prior experience of these medicines.

Public initiatives are needed to expand medical education within healthcare settings, including public and private healthcare settings. Educational initiatives and campaigns should target healthcare providers and diabetic patients. These campaigns should clarify disease misinformation. Decision makers should regulate the use of GLP-1 RAs at the national level to promote the safe and effective usage of these medications.

Healthcare providers must employ varied means of communication, such as social media, to offer accurate and sufficient information about GLP-1 receptor agonists so that patients can take informed decisions for themselves. Later studies can investigate other determinants influencing public knowledge and perception, such as healthcare provider advice, cultural determinants, and patients’ use of digital health resources. Moreover, further research must investigate the long-term compliance problems of GLP-1 RAs and potential ways to enhance patient retention and treatment success.

This study has limitations. The use of an online survey study design and recruiting the participants using a convenience sampling technique might have affected the generalizability of the study findings. This methodological approach might only represent participants who have access to online platforms. However, due to the widespread use of social media platforms in Saudi Arabia across all demographic groups, we expect that the impact of this methodological approach on the generalizability of our findings is minimal. Therefore, the study findings should be interpreted carefully.

## 5. Conclusion

The findings of this study indicate that participants have a moderate to high level of awareness of GLP-1 enhancers, with Mounjaro being the most well-known brand and social media serving as the primary source of information. The majority of individuals reported that they used these medications predominantly for weight reduction, and they frequently experienced side effects such as abdominal pain, vomiting, and appetite loss. Notably, individuals in lower income brackets exhibited substantially better knowledge of GLP-1 enhancers than those in higher income brackets, while prior or current users exhibited the highest levels of awareness, highlighting the important role of clinical exposure. Future research should investigate the fundamental causes of income-related disparities in knowledge and evaluate the effectiveness of social media as an educational resource. Moreover, future research should examine the impact of educational interventions on participants’ awareness and safety perceptions. Furthermore, longitudinal studies are required to assess the long-term safety, efficacy, and awareness of GLP-1 enhancers in a variety of populations.

## Author contributions

**Conceptualization:** Amal Khaleel AbuAlhommos.

**Data curation:** Amal Khaleel AbuAlhommos.

**Formal analysis:** Amal Khaleel AbuAlhommos.

**Funding acquisition:** Amal Khaleel AbuAlhommos.

**Investigation:** Amal Khaleel AbuAlhommos, Maitham Abdullah Al Hawaj, Sharifa Yousef Alrasheed, Reem Abdulrahman Alessa, Alzahra Yousef Alhussain, Rouaa Adel Alabdulkareem, Raghad Ibrahim Abdulaziz Alzamil.

**Methodology:** Amal Khaleel AbuAlhommos.

**Project administration:** Amal Khaleel AbuAlhommos, Maitham Abdullah Al Hawaj.

**Resources:** Amal Khaleel AbuAlhommos, Maitham Abdullah Al Hawaj, Sharifa Yousef Alrasheed, Reem Abdulrahman Alessa, Alzahra Yousef Alhussain, Rouaa Adel Alabdulkareem, Raghad Ibrahim Abdulaziz Alzamil.

**Software:** Amal Khaleel AbuAlhommos.

**Supervision:** Amal Khaleel AbuAlhommos.

**Validation:** Amal Khaleel AbuAlhommos.

**Visualization:** Amal Khaleel AbuAlhommos.

**Writing – original draft:** Amal Khaleel AbuAlhommos.

**Writing – review & editing:** Amal Khaleel AbuAlhommos, Maitham Abdullah Al Hawaj, Sharifa Yousef Alrasheed, Reem Abdulrahman Alessa, Alzahra Yousef Alhussain, Rouaa Adel Alabdulkareem, Raghad Ibrahim Abdulaziz Alzamil.
